# Next-generation adjuvant systems containing furfurman drives potent adaptive immunity and host defense as a foot-and-mouth disease vaccine adjuvant

**DOI:** 10.3389/fimmu.2024.1491043

**Published:** 2024-12-11

**Authors:** Hyeong Won Kim, Seokwon Shin, So Hui Park, Jong-Hyeon Park, Su-Mi Kim, Yoon-Hee Lee, Min Ja Lee

**Affiliations:** Center for Foot-and-Mouth Disease Vaccine Research, Animal and Plant Quarantine Agency, Gimcheon-si, Gyeongsangbuk-do, Republic of Korea

**Keywords:** foot-and-mouth disease, furfurman, vaccine adjuvant, innate and adaptive immunity, host defense

## Abstract

**Introduction:**

Many countries use commercial foot-and-mouth disease (FMD) vaccines to prevent FMD pandemics, but these vaccines have disadvantages, such as repeated vaccinations due to the short persistence of antibody (Ab) titers and incomplete host defense despite high Ab titers. To address these shortcomings, we aimed to develop a novel FMD vaccine containing furfurman as an adjuvant.

**Method:**

To demonstrate the efficacy of the test vaccine, adaptive immunity was evaluated by measuring Ab and neutralizing Ab titers and host defense against viral infections in experimental and target animals. In addition, the expression levels of cytokines [interferon (IFN)α, IFNβ, IFNγ, interleukin (IL)-1β, IL-2, and IL-12p40] were evaluated at the early stages of vaccination to confirm the simultaneous induction of cellular and humoral immune responses induced by the test vaccine.

**Result:**

The groups that received vaccine containing furfurman showed a strong early, mid-term, and long-term immune response and host defense against viral infections compared to the control groups. The significant upregulation observed in cytokine levels in the furfurman group compared to those in the control groups strongly suggest that the test vaccine strengthens cellular immune response and effectively induces a humoral immune response.

**Conclusion:**

Our study demonstrated that furfurman, as an FMD vaccine adjuvant, achieves long-lasting immunity and host defense against viral infections by eliciting potent cellular and humoral immune responses. Therefore, our findings contribute to the design of next-generation FMD vaccines and highlight the potential application of furfurman as an adjuvant for other viral diseases.

## 1 Introduction

Foot-and-mouth disease (FMD) is a highly contagious viral disease that affects livestock, such as pigs, goats, and cattle. FMD spreads primarily through aerosols, and symptoms can appear two to three days after infection in animals. Typical clinical symptoms of FMD infection include blisters, drooling, loss of hooves, and death in young animals (1). The FMD virus (FMDV) belongs to the *Picornaviridae* family and is broadly divided into seven serotypes with numerous subtypes. It is difficult to control FMD because cross-protection between the seven FMDV serotypes is not possible (2).

Majority of the commercially available FMD vaccines that are currently being used to prevent FMD pandemics are inactivated FMD vaccines, which use inactivated whole FMDV as antigens. Commercial FMD vaccines have major drawbacks, such as low antibody (Ab) titers in pigs compared to those in cattle, short persistence of Ab titers, and incomplete humoral immunity-mediated host defense (3). To overcome these shortcomings, we used furfurman as an FMD vaccine adjuvant. In a previous study, FMD vaccines containing various pattern recognition receptor (PRR) ligands elicited high Ab and virus-neutralizing (VN) titers in pigs (4). Furfurman is a dendritic cell (DC)-associated C-type lectin-2 (dectin-2) agonist and C-type lectin-like receptor (CTLR) that stimulates the secretion of Th17 cytokines, such as interleukin (IL)-23, IL-17A, and IL-6 (5). CTLRs such as dectin-1, dectin-2, and Mincle stimulate the innate immune system of host. Among the many CTLR family members, those that are critical for stimulating innate and adaptive immunity include dectin-1 and dectin-2 (6). The well-known function of CTLR is to uptake antigens and present them to T cells. Another function is that it stimulates downstream NF-κB signaling pathways and then leads to the secretion of inflammatory cytokines, which then contributes to innate and adaptive immunity (7–9). Dectin-2 is expressed on many immune cells, such as neutrophils, macrophages (MΦs), and DCs (10, 11). Downstream signaling of dectin-2 promotes the secretion of cytokines (IL-1β, IL-2, IL-12, and IL-23) (12).

We hypothesized that an advanced FMD vaccine that uses furfurman as an adjuvant could address the shortcomings of commercial vaccines. In addition to furfurman, the test vaccine contained various adjuvants, such as alum, Quil-A, and ISA206. Alum is adsorbed onto the antigen, maintains its durability, and induces long-term immunity by prolonging the interaction period between the antigen and immune cells (13). Quil-A has the advantages of low toxicity and a simpler formulation than crude saponin adjuvants (14). As a viral vaccine adjuvant, Quil-A enhances the expression of type I and type II IFNs (15). ISA206, an oil-based adjuvant, has the advantage of inducing a long-term immune response; however, it can cause local side effects when used at high doses in vaccines (16, 17). ISA206 enhances humoral immunity and upregulates the production of cytokines such as IL-2 and IFNγ (18, 19). In the present study, we assessed a vaccine containing furfurman that can mediate cell proliferation *in vitro* [peritoneal exudate cells (PECs) in mice and peripheral blood mononuclear cells (PBMCs) in pigs] and assessed how it influences early, mid-term, and long-term immune responses and host defense against FMDV type O and type A infections *in vivo*. We also evaluated cytokine expression to verify whether the test vaccine containing furfurman could induce cellular immune responses.

## 2 Materials and methods

### 2.1 Animals

Mice (C57BL/6, 6–7 weeks old, female) and farm pigs (Landrace; 8–9 weeks old) were purchased from KOSA BIO Inc. (Gyeonggi-do, Republic of Korea) and BARON BIO Inc. (Gyeongsangbuk-do, Republic of Korea), respectively. Mice and pigs were housed in a specific pathogen-free animal biosafety level 3 facility at the Animal and Plant Quarantine Agency (4). This study was approved by the Ethics Committee of the Animal and Plant Quarantine Agency (certification nos.: IACUC-2022-670 and 2023-753).

### 2.2 Cells and viruses

LF-BK (fetal porcine kidney cell line), ZZR 127 (fetal goat tongue epithelium), and BHK-21 (baby hamster kidney) cells, as well as FMDV O PA2 (GenBank accession no. AY593829.1) and FMDV A YC (GenBank accession no. KY766148.1) (4), were used in this study. Viruses were cultured in Dulbecco’s Modified Eagle’s medium (HyClone, Logan, UT, USA).

### 2.3 Antigen purification

BHK-21 cell line was used for antigen production. FMDV (O PA2 and A YC) was used as a source to produce inactivated antigens. Sixteen hours after infection, the viruses were inactivated using two doses of binary ethyleneimine (0.003 N). The inactivated viruses were concentrated through polyethylene glycol 6000 (Sigma-Aldrich, Waltham, MO, USA) treatment. Antigens were purified using the sucrose density gradient method (15–45%) and thereafter ultracentrifuged. After ultracentrifugation, the bottom of the centrifuge tube was punctured, and 1 mL fractions were collected. The presence of FMDV particles in the sample of each fraction was confirmed using a lateral flow device (BioSign FMDV Ag; Princeton BioMeditech, NJ, USA) (20). The 146S antigen was quantified using spectrophotometry at 259 nm. An inactivation test using ZZR 127 and BHK-21 cells confirmed that no live viruses were present in the inactivated supernatants (21).

### 2.4 Composition and preparation of test vaccine

In the mouse experiment, the vaccine composition for the positive control (PC) group was as follows: purified antigen types O (O PA2; 0.375 μg/dose) and A (A YC; 0.375 μg/dose), 15 μg/dose Quil-A (InvivoGen, San Diego, CA, USA), 10% alum, and ISA206 (Seppic, Paris, France; 50% w/w). The experimental (Exp) group received vaccines with the same composition as that of the PC group, with the addition of 100 μg furfurman/dose. One dose comprised a total volume of 100 μL. The negative control (NC) group received the same volume of phosphate-buffered saline (PBS; Gibco, Grand Island, NY, USA).

In the pig experiment, the vaccine formula for the PC group was as follows: purified antigen types O (15 μg/dose) and A (15 μg/dose), 150 μg/dose Quil-A, 10% alum, and ISA206 (50% w/w). The Exp group received test vaccines with the same formula as that of the PC group, with the addition of 1 mg furfurman/dose. One dose comprised a total volume of 1 mL. The NC group received the same volume of PBS.

Inactivated antigens (O PA2 and A YC) were mixed (adjuvanted) with 10% alum in an ice and left to stand for 1 h to adsorb and create a depot. After dispensing Quil-A and furfurman into the suspension (aqueous phase) containing antigen and 10% alum, the weight of the aqueous layer was adjusted with TK buffer (Tris-KCl; pH 7.4). After dispensing ISA206 (50% w/w), homogenization was performed at low speed (1,200 rpm) with ultrahomogenizer in an ice bath according to the manufacturer’s instructions. Emulsification was performed until a milky, low viscous and stable emulsion was obtained. The prepared vaccine was stored at 4°C until vaccination of animals and regularly monitored for stability and immunogenicity.

### 2.5 Peritoneal exudate and peripheral blood mononuclear cell isolation

Naive mice (*n* = 10) were euthanized, following a previously described experimental protocol (21). The abdominal cavity was washed with PBS, and the peritoneal lavage fluid was centrifuged. Whole blood (10 mL/donor) from pigs (*n* = 5–6/group) was used, as previously described (21). PBMCs were isolated with Lymphoprep (Stem Cell Technologies, Vancouver, Canada). Red blood cells were removed using ammonium–chloride–potassium lysing buffer (Gibco). The collected PECs and PBMCs were counted with a cell counter (Bio-Rad TC20; Bio-Rad Laboratories, Hercules, CA, USA). The isolated cells were cultured in RPMI-1640 medium (Gibco). All cells were used immediately after isolation.

### 2.6 BrdU incorporation assay

The proliferation of PECs and PBMCs was assessed using a BrdU cell proliferation assay kit (Cell Signaling Technology, Danvers, MA, USA) (4), following the manufacturer’s guidelines. The test vaccines (with or without furfurman) were administered to fresh PECs and PBMCs, and the results were confirmed at 0, 6, 12, and 24 h.

### 2.7 Serological assays

To evaluate the structural protein (SP) Abs in sera, PrioCheck FMDV type O and type A kits (Prionics AG, Schlieren, Switzerland) were used, according to the manufacturer’s instructions (21). Absorbance was measured at a wavelength of 450 nm and converted to percentage inhibition (PI) values. For the PrioCheck FMDV kit, a PI value ≥50% was considered Ab-positive.

VN test was performed according to the protocols specified by the World Organization for Animal Health (22, 23). Briefly, serum was heat-inactivated and then diluted. Thereafter, 50 μL TCID_50_ FMDV (O PA2 or A YC) was added and incubated for 1 h. A 50 μL volume of LF-BK cells (10^4^ cells/well) was added to each well and cultured for three days. Subsequently, cytopathic effects were confirmed in each well (21, 24).

Enzyme linked immunosorbent assays (ELISAs) were performed using kits for porcine IFNα, IFNβ, IFNγ, IL-1β, IL-2, and IL-12p40 (DuoSet, R&D Systems, Minneapolis, MN, USA; Cloud-Clone Corp Inc., Houston, TX, USA) according to the manufacturer’s guidelines.

### 2.8 Evaluation of early, mid-term, and long-term immunity and host defense in mice immunized with the test vaccine

Early, mid-term, and long-term immune responses to the test vaccine were evaluated in mice (*n* = 5/group) using a previously described experimental protocol (21). Host defense against viral infections was assessed in mice vaccinated with the test vaccine. Mice were vaccinated via intramuscular injections (0 dpv) and challenged with the FMDV [100 lethal dose 50% (LD_50_) O/VET/2013 or 100 LD_50_ A/Malay/97] via intraperitoneal injections at 7, 28, 84, and 168 dpv. Survival rates and body weights were monitored for up to 7 d post-challenge (dpc).

### 2.9 Evaluation of early, mid-term, and long-term immunity in pigs immunized with the test vaccine

Early, mid-term, and long-term immune responses to the test vaccine were evaluated in pigs (*n* = 5–6/group) using a previously described experimental protocol (21). For serological analysis, sera were collected from the vaccinated pigs at 0, 7, 14, 28, 56, and 84 dpv. After the first vaccination, a second vaccination was performed at 28 dpv using the same route.

### 2.10 Evaluation of host defense against FMDV infection in pigs after administration of the test vaccine

To evaluate whether the test vaccine could induce host defense, a challenge experiment was performed (*n* = 3/group). At 28 dpv, all experimental groups were infected with FMDV types O and A (10^5^ TCID_50_/100 μL) via intradermal injections into the soles of the feet. Observation of clinical symptoms and collection of oral swab samples (BD Universal Viral Transport Kit; BD Biosciences, Franklin Lakes, NJ, USA) were performed daily during the challenge period. Serum (vacutainer serum tubes; BD Biosciences) was collected at 0, 2, 4, 6, and 8 dpc. RNA was extracted from oral swabs and serum, according to the instructions of the QIAcube HT Pathogen Kit (QIAGEN, Leipzig, Germany). RT-PCR was conducted using the FMDV Real-Time RT-PCR Master Mix Kit (Bioneer, Daejeon, Republic of Korea), according to the manufacturer’s instructions (21).

### 2.11 RNA extraction, cDNA synthesis, and quantitative RT-PCR

RNA was extracted using the RNeasy Mini Kit (QIAGEN) and TRIzol reagent (Invitrogen, Carlsbad, CA, USA), according to the manufacturer’s guidelines. The cDNA was synthesized using the GoScript Reverse Transcription System (Promega, Madison, WI, USA), according to the manufacturer’s guidelines. Afterwards, quantitative RT-PCR (qRT-PCR) was performed using SYBR Green Supermix (Bio-Rad) (21). The qRT-PCR results were normalized using the measured *hprt* (reference gene) levels. The primers used are shown in **Supplementary Table 1**.

### 2.12 Statistical analysis

Unless otherwise specified, all data are presented as the mean ± SEM. Survival curves were drawn using the Kaplan–Meier method, and differences were analyzed using the log-rank sum test. Statistical differences between groups were determined using Tukey’s or Dunnett’s *post-hoc* tests and one-way or two-way analysis of variance. Statistical significance is indicated by ^*^ *p <*0.05, ^**^ *p <*0.01, ^***^ *p <*0.001, and ^****^ *p <*0.0001. All data were analyzed using GraphPad Prism 10.2.3 (GraphPad, San Diego, CA, USA).

## 3 Results

### 3.1 Test vaccine containing furfurman stimulated the proliferation of immune cells

**Figure 1:**
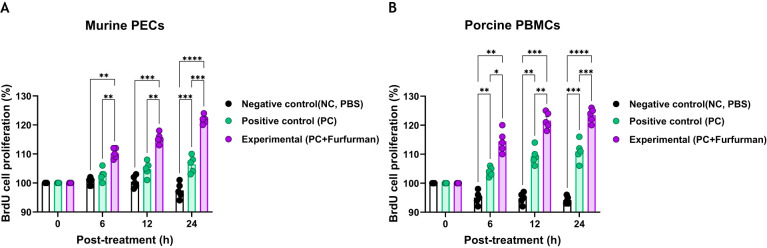
Vaccine containing furfurman induces cell proliferation in murine PECs and porcine PBMCs. The level of cell proliferation, as measured using a BrdU cell proliferation kit, was assessed 6, 12, and 24 h after murine PECs and porcine PBMCs were treated with the test vaccine **(A, B)**. BrdU cell proliferation in murine PECs **(A)** and porcine PBMCs **(B)**. Statistical analyses were performed using one-way ANOVA, followed by Tukey’s *post-hoc* test. *p<0.05; **p<0.01; ***p<0.001; and ****p<0.0001. PBMC, peripheral blood mononuclear cell; PEC, peritoneal exudate cell.

To investigate the effect of test vaccine containing furfurman on immune responses, cell proliferation via the BrdU assay was assessed 6, 12, and 24 h after murine PECs and porcine PBMCs were treated with the test vaccine (**Figures 1A, B**). At all-time points (6, 12, and 24 h) measured for the murine PECs, the Exp group showed higher cell proliferation rates than the PC group (**Figure 1A**). Similarly, porcine PBMCs showed a significantly higher cell proliferation rate in the Exp group than in the PC group at all-time points (**Figure 1B**). These results demonstrate that furfurman stimulates the host innate immune response, indicating its potential use as an adjuvant.

### 3.2 Test vaccine containing furfurman elicited potent and long-lasting humoral immune responses in mice

**Figure 2:**
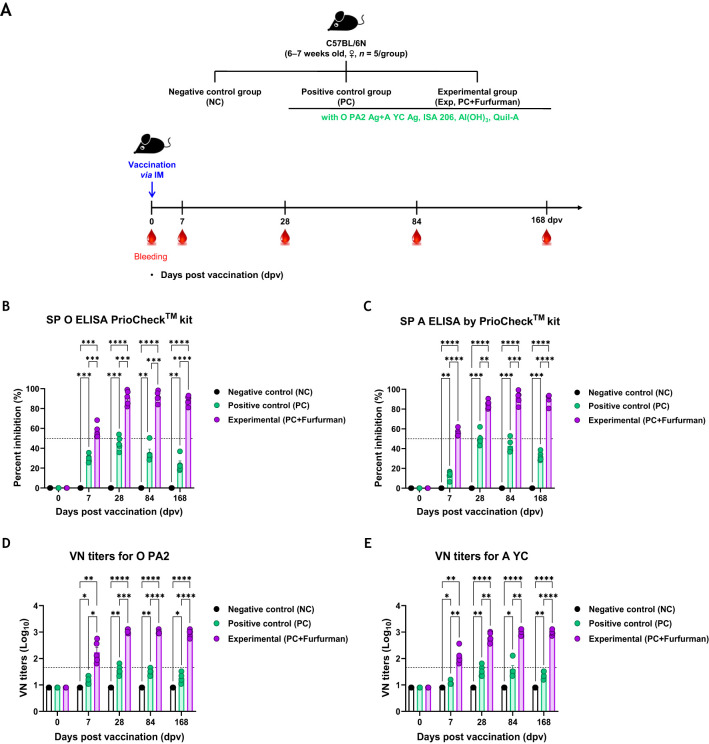
Vaccine containing furfurman elicits potent cellular and humoral immunity, leading to robust long-lasting humoral immune responses. C57BL/6 mice were administered the test vaccine with (Exp group) or without (PC group) furfurman. Mice were vaccinated with the test vaccine via the intramuscular (IM) route, and blood was then collected at 0, 7, 28, 84, and 168 d post-vaccination (dpv) for serological analysis using structural protein (SP) O and A ELISA kits and virus-neutralizing (VN) titers for O/PKA/44/2008 (O PA2) and A/SKR/YC/2017 (A YC). **(A–E)** Experimental strategy **(A)**; antibody titers, as determined using SP O **(B)** and SP A **(C)** ELISA kits; VN titers for O PA2 **(D)** or A YC **(E)**, as determined using VN tests. Data are represented as the mean ± SEM of triplicate measurements (*n* = 5/group). Statistical analyses were performed using two-way ANOVA, followed by Tukey’s *post-hoc* test. ^*^ *p <*0.05; ^**^ *p <*0.01; ^***^ *p <*0.001; and ^****^ *p <*0.0001.

To evaluate long-lasting humoral immune responses of mice to the test vaccines, experiments were conducted according to the design depicted in **Figure 2A**. The Exp group had higher Ab titers specific to the antigens (types O and A) than the PC group in all aspects, including the rate of increase, maximum value, and sustainability of Ab titers, as measured via SP ELISA (**Figures 2B, C**). VN titers for FMDV types O and A were also significantly higher in the Exp group than in the control group at all-time points. Notably, VN titers in the Exp group tended to increase rapidly and remained constant for a long period (**Figures 2D, E**). These results demonstrate that furfurman elicits potent adaptive immunity as an FMD vaccine adjuvant, leading to rapid and robust long-term immunity.

### 3.3 Test vaccine containing furfurman induced broad-duration range of host defense against viral infection in mice

**Figure 3:**
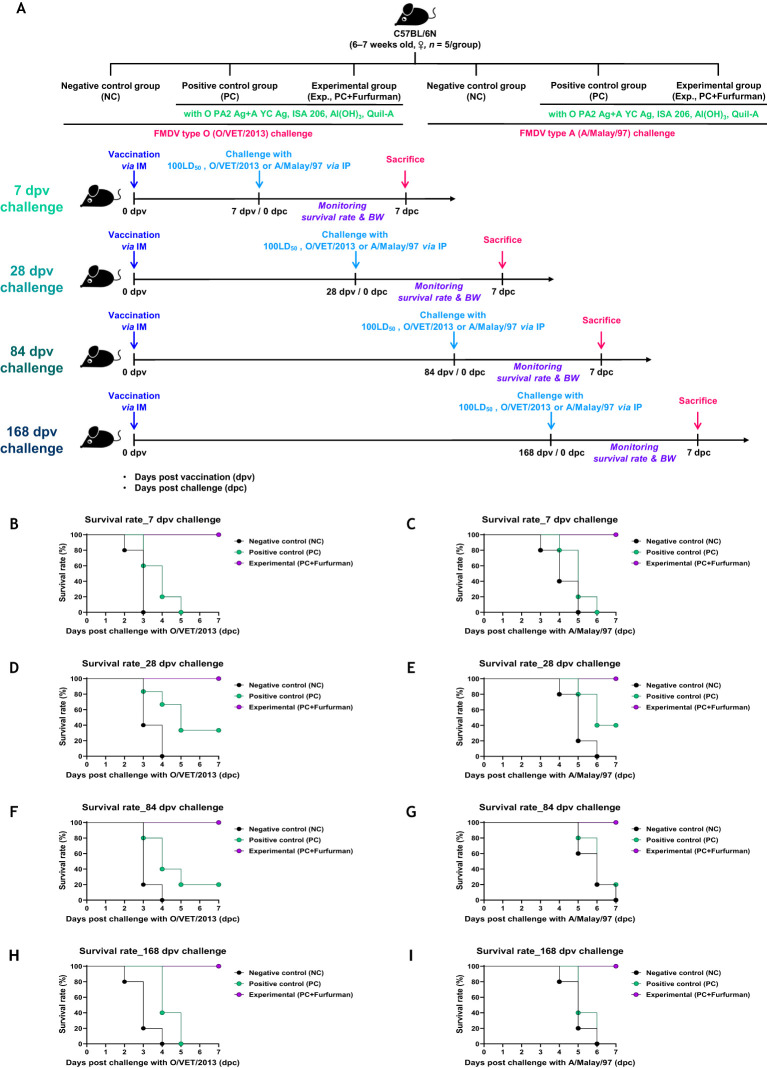
Vaccine containing furfurman drives potent host defense against viral infection. C57BL/6 mice were administered test vaccine with (Exp group) or without (PC group) furfurman. The test vaccines were injected via the intramuscular (IM) route into mice that were later challenged with foot-and-mouth disease virus (FMDV) O (100 lethal dose 50% [LD_50_] O/VET/2013) or FMDV A (100 LD_50_ A/Malay/97) at 7, 28, 84, and 168 d post-vaccination (dpv) via the intraperitoneal (IP) route. Survival rates and body weights were monitored for 7 d post-challenge (dpc). **(A–I)** Experimental strategy **(A)**; survival rates post-challenge with O/VET/2013 **(B)** and A/Malay/97 **(C)** at 7 dpv; survival rates post-challenge with O/VET/2013 **(D)** and A/Malay/97 **(E)** at 28 dpv; survival rates post-challenge with O/VET/2013 **(F)** and A/Malay/97 **(G)** at 84 dpv; survival rates post-challenge with O/VET/2013 **(H)** and A/Malay/97 **(I)** at 168 dpv. Data are presented as the mean ± SEM of triplicate measurements (*n* = 5/group).

To investigate the efficacy of the test vaccine in host defense against viral infection, experiments were conducted according to the design depicted in **Figure 3A**. In the challenge experiment (FMDV types O and A) at 7 dpv after immunization with the test vaccine, the Exp group showed a 100% survival rate at 7 dpc, whereas the PC group showed a 0% survival rate (**Figures 3B, C**). In the challenge experiments conducted at 28, 84, and 168 dpv, the Exp group showed a 100% survival rate throughout, whereas the PC group showed a 40%, 20%, and 0% survival rate, respectively (**Figures 3D–I**). There were no significant differences observed in body weight between groups under all conditions tested (**Supplementary Figure 1**). These results demonstrate that the test vaccine elicited a robust host defense against FMDV infection during the early (7 dpv), mid-term (28 dpv), and long-term (84 and 168 dpv) periods.

### 3.4 Test vaccine containing furfurman induced long-lasting immunity in pigs

**Figure 4:**
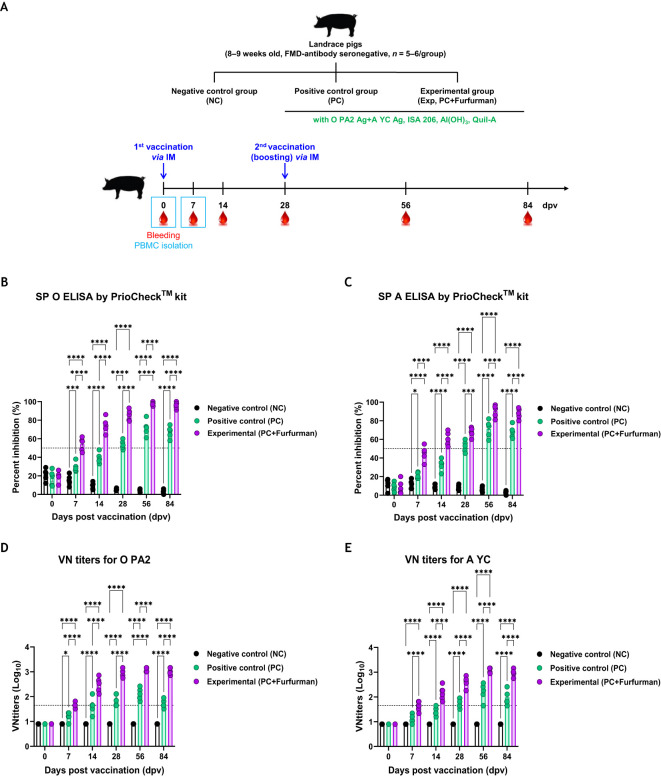
Vaccine containing furfurman elicits potent humoral immune responses in pigs. Landrace pigs were divided into three groups (*n* = 5–6/group) and administered the test vaccine with (Exp group) or without (PC group) furfurman. Vaccination was performed twice at 28-d intervals, with 1 mL vaccine (one dose) injected via the deep intramuscular (IM) route into the necks of the animals. The NC group was injected with an equal volume of PBS. Blood samples were collected from pigs at 0, 7, 14, 28, 56, and 84 d post-vaccination (dpv) for serological assays. **(A–E)** Experimental strategy **(A)**; antibody titers, as determined using structural protein (SP) O **(B)** and SP A **(C)** ELISA kits; virus-neutralizing (VN) titers for O PA2 **(D)** or A YC **(E)**, as determined using VN tests. Data are represented as the mean ± SEM of triplicate measurements (*n* = 5–6/group). Statistical analyses were performed using two-way ANOVA, followed by Tukey’s *post-hoc* test. ^*^ *p <*0.05; ^***^ *p <*0.001; and ^****^ *p <*0.0001.

Experiments were performed to assess how adaptive and long-lasting immunity was affected by the test vaccine in pigs, as shown in **Figure 4A**. The Exp group had higher Ab titers specific to the antigens (types O and A) than the PC group for all types from 7–84 dpv, as measured via SP ELISA (**Figures 4B, C**). VN titers against FMDV types O (O PA2) and A (A YC) increased more rapidly in the Exp group than in the PC group, and their maximum VN titers was also higher. Overall, the VN titers were significantly higher in the Exp group than in the PC group from 7–84 dpv (**Figures 4D, E**). These results demonstrate that the test vaccine elicited long-lasting immunity depending on the stimulation of cellular and humoral immunity in pigs.

### 3.5 Test vaccine containing furfurman enhanced immune response through regulation of cytokine expression

**Figure 5:**
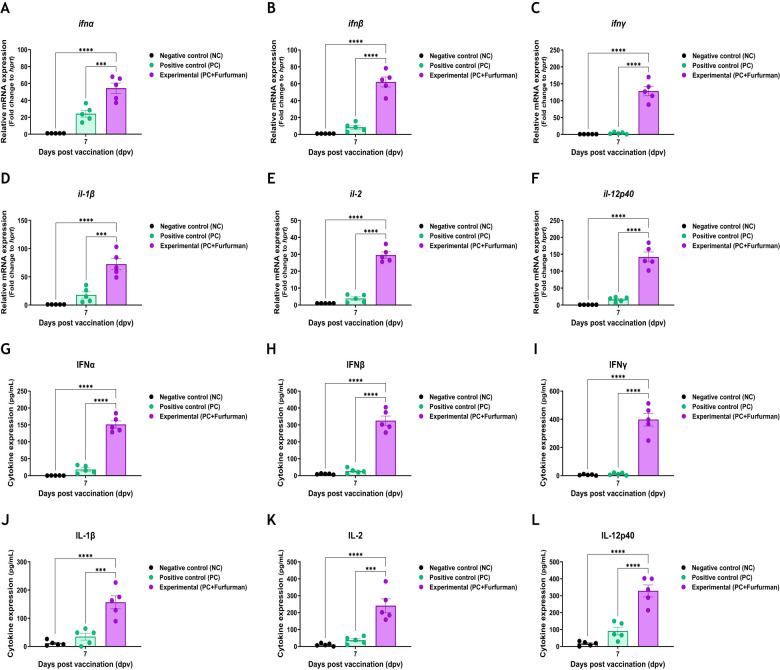
Vaccine containing furfurman induces the expression of proinflammatory cytokines in pigs. Porcine peripheral blood mononuclear cells (PBMCs) and serum isolated from the whole blood of vaccinated pigs (*n* = 5–6/group), as described in **Figure 4A**, were used for quantitative RT-PCR and ELISA. Gene expression levels were normalized to those of *hprt* and presented as relative ratios when compared to the gene expression levels of the control. **(A–L)** Gene expression levels of *ifnα* **(A)**, *ifnβ* **(B)**, *ifnγ* **(C)**, *il-1β* **(D)**, *il-2* **(E)**, and *il-12p40* **(F)**; protein secretion levels of IFNα **(G)**, IFNβ **(H)**, IFNγ **(I)**, IL-1β **(J)**, IL-2 **(K)**, and IL-12p40 **(L)**. Statistical analyses were performed using two-way ANOVA, followed by Tukey’s *post-hoc* test. ^***^ *p <*0.001 and ^****^ *p <*0.001.

To understand the background of the test vaccine-mediated potent immune response, RNA and protein levels of IFNα, IFNβ, IFNγ, IL-1β, IL-2, and IL-12p40 were assessed via qRT-PCR and ELISA during the early stage of post vaccination. This experiment was performed using PBMCs derived from whole blood and serum, as shown in **Figure 4A**. Gene expression levels of cytokines were higher in the Exp group than in the PC group (**Figures 5A−F**). Similar to the results of the gene expression levels, the protein expression levels of cytokines in the Exp group were significantly higher than those in the PC group (**Figures 5G−L**). These results demonstrate that the test vaccine rapidly stimulated immune cells and elicited potent cellular and humoral immunity during the early stages of vaccination.

### 3.6 Test vaccine containing furfurman induced robust host defense against FMDV infection in pigs

**Figure 6:**
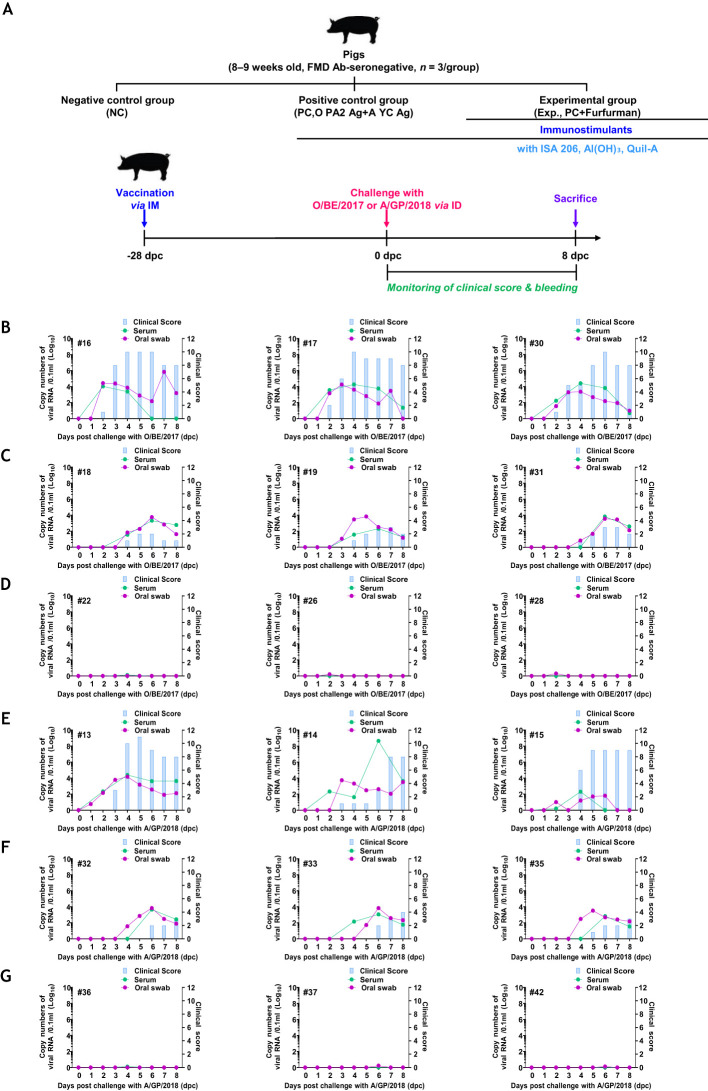
Vaccine containing furfurman drives robust host defense against FMDV infection in pigs. Landrace pigs were divided into three groups (*n* = 3/group) and administered the test vaccine with (Exp group) or without (PC group) furfurman. Blood samples were collected at 0 and 28 d post-vaccination (dpv) for serological assays. Vaccinated pigs were challenged with foot-and-mouth disease virus (FMDV) types O and A (O/SKR/BE/2017, A/SKR/GP/2018; 10^5^ TCID_50_/100 μL) via intradermal injection on the heel bulb at 28 dpv. **(A–G)** Experimental workflow **(A)**; clinical score and viral load (titers) of serum samples and oral swabs from the NC **(B)**, PC **(C)**, and Exp groups **(D)** infected with FMDV type O; clinical score and viral load of serum samples and oral swabs from the NC **(E)**, PC **(F)**, and Exp groups **(G)** infected with FMDV type **(A)** The left Y-axis of the graph shows the amount of virus in the serum and oral swab samples, represented as Log_10_ values, whereas the right Y-axis shows the clinical index as the maximum value of 10 points. Data are presented as the mean ± SEM of triplicate measurements (*n* = 3/group).

To assess host defense against FMDV (types O and A) infection in pigs immunized with the test vaccine containing furfurman, a target animal challenge was performed, as described in **Figure 6A**. The NC group failed to achieve host defense and showed typical clinical symptoms of FMD against both types of FMDV infections. Additionally, high viremia was observed in the serum and oral swabs (**Figures 6B, E**). The PC group showed fewer clinical symptoms of FMD than the NC group, but high viremia was observed in the serum and oral swabs, and this is similar to that in the NC group, which failed to demonstrate complete host defense against viral infection (**Figures 6C, F**). In contrast, in the Exp group, which received the test vaccine containing furfurman, no clinical symptoms of FMD were observed, and viremia was neither observed in the serum nor in oral swabs (**Figures 6D, G**). These results highlight the strong adjuvanticity of furfurman in the test vaccine and demonstrate that the test vaccine elicited a potent host defense mechanism against FMDV infections.

## 4 Discussion

In human vaccines, various adjuvants, such as immunostimulants (e.g., PRR ligands, cytokines, and small molecules) and antigen-delivery systems [e.g., lipid nanoparticles, polymeric particles (poly lactic-co-glycolic acid), caged protein nanoparticles, and inorganic nanocarriers], have been studied, and clinical trials are in progress or partially performed (25, 26); however, their use is limited in animal vaccines. The FMD vaccine contains several adjuvants (e.g., oil-based emulsion, saponin, and aluminum gel), along with an antigen, to enhance the sustainability of vaccine efficacy through antigen stability and slow release. However, oil-based adjuvants have the disadvantage of causing local side effects because the oil adjuvant clumps at the injection site. In particular, oil-based adjuvants induce humoral immunity, making it difficult to induce cellular immunity. It takes a certain period of time to induce Ab titers to a defensive level, making initial protection difficult (27). To overcome these shortcomings and improve vaccine efficacy, we designed a test vaccine using furfurman as an FMD vaccine adjuvant and evaluated its efficacy.

The importance of CTLR (dectin-1, dectin-2, Mincle, and DC-SIGN) and cGAS-STING pathway-mediated innate immunity in host–pathogen interactions has been emphasized previously (25, 28). Furfurman is a dectin-2 agonist that induces a potent immune response against invaders (pathogens including viruses and bacteria) within the host. Dectin-2 stimulation not only elicits mucosal immunity by inducing secretory IgA production but also promotes phagocytosis, thereby contributing to innate immunity (5, 29). Using an adjuvant that simultaneously induces systemic and mucosal immunity could maximize host immune-boosting efficacy through a synergistic effect (30). Currently, the use of PRR agonists as vaccine adjuvants is the latest trend (28). As a vaccine adjuvant, furfurman simultaneously induces systemic and mucosal immunity.

PECs and PBMCs contain antigen-presenting cells, such as DCs and MΦs, as well as lymphocytes (T, B, and NK cells) (31). Previous studies have evaluated the proliferation of pig PBMCs 96 hrs after administration of vaccines containing furfurman (4). However, in this study, furfurman rapidly stimulated and induced proliferation of mouse and pig immune cells within 24 hrs. Therefore, we concluded that FMD vaccines containing furfurman could induce a potent immune response in the early stage (**Figure 1**). Based on these results, we evaluated the adjuvanticity of furfurman and efficacy [adaptive (early, mid-term, and long-term) immunity] of a furfurman-containing vaccine in mice. The Exp group immunized with the test vaccine maintained significant Ab and VN titers compared to those in the PC group until 168 dpv. During the process of developing long-term immunity, the test vaccine not only showed a faster rate of Ab titer accumulation than the PC group did but also allowed the elevated Ab titers to persist for a longer period of time (**Figure 2**). Ab and VN titers are key indicators of adaptive (humoral) immunity (32). Therefore, the test vaccine likely elicited long-term immunity by inducing adaptive immunity. During early, mid-term, and long-term host defense in mice, the Exp group exhibited 100% protection at all time points measured, whereas the PC group exhibited 40% and 20% survival rates at 28 and 84 dpv, respectively. As shown in **Figure 2**, the PC group had the highest Ab and VN titers at 28 dpv but gradually decreased thereafter, transitioning to Ab-negative levels at 168 dpv (**Figure 3**).

Similar to the previous results observed in mice, pigs immunized with the test vaccine reached Ab-seropositive levels at 7 dpv. VN titers in the Exp group were significantly different from those in the PC group. In addition, pigs in the Exp and PC groups exhibited significant differences in both Ab and VN titers until 84 dpv (**Figure 4**). These results demonstrate that the test vaccine containing furfurman addressed the limitation of the existing FMD vaccine (short Ab titer maintenance period) (33) and elicited long-lasting immunity based on the induction of potent cellular and humoral immunity. Unlike the other control groups, the Ab and VN titers of pigs immunized with the test vaccine significantly increased in the early stages (7 dpv) (**Figure 4**).

To elucidate the background of this robust host immune response, we assessed the level of key cytokines (IFNα, IFNβ, IFNγ, IL-1β, IL-2, and IL-12p40) that induce innate and adaptive immunity at the gene and protein levels. The Exp group immunized with the test vaccine showed significantly higher expression in all the investigated cytokines at the gene and protein levels than the PC group. Type I IFNs (IFNα and IFNβ) are the first line of host defense produced during the viral infection and contribute to the induction of innate immune responses (34). Type I IFNs respond to infections by stimulating the innate and adaptive immunity of host during pathogen infection (35, 36). Specifically, type I IFNs not only elicit host immune responses by stimulating NK, T, B, and myeloid cells but also induce memory responses to prevent secondary viral infection. Therefore, during host infection, viruses suppress the expression of type I IFNs through their own mechanisms (37–40). IFNγ is secreted by immune-related cells, including gamma delta T, NKT, T, B, and NK cells (41–44). IFNγ regulates host innate and adaptive immunity through the induction of potent cellular immunity (45, 46). IFNγ downregulates inhibitory cytokines (IL-10) and promotes IL-12 production in MΦs (47, 48). IL-1β, a proinflammatory cytokine, is a critical marker of the inflammatory response that stimulates the immune response of host (49). IL-1β is produced through cleavage of pro-IL-1β by activated inflammasomes (50) and secreted by cells involved in innate and adaptive immunity stimulated through inflammatory signals (51). IL-1β contributes to enhancing innate immunity and inducing adaptive immunity (52, 53). IL-2 stimulates T cell proliferation and memory cell production, leading to a potent adaptive immune response in the host (54). Moreover, IL-2 regulates the expression of transcription factors and cytokines in CD4^+^ T cells, controlling their maturation toward the Th1 and Th2 phenotypes (55–58). IL-2 also controls the differentiation and maturation of CD8^+^ T cells. High IL-2 production induces differentiation of CD8^+^ T cells into CD8^+^ cytotoxic T cells (59). In contrast, low IL-2 production promotes the differentiation of CD8^+^ memory cells (60–62). IL-12p40 secreted by DCs contributes to T cell-mediated immunity by eliciting IL-12 secretion (63, 64). IL-12 is mainly induced by IFNγ, both of which form a positive feedback loop that promotes their own secretion (65, 66). As with IFNγ, IL-12 induces innate and adaptive immunity. Furthermore, IL-12 is activated via TLR stimulation and secreted by MΦs, monocytes, DCs, and B cells (67, 68). The main functions of IL-12 include activating antigen presentation and enhancing the cytolysis of NKT and NK cells (48, 69). Overall, the results indicate that the test vaccine stimulated immune cells during the early stages of vaccination, eliciting robust innate and adaptive immunity based on potent cellular and humoral immunity (**Figure 5**).

Finally, a challenge experiment was performed in pigs to verify whether those immunized with the test vaccine had host defenses against FMDV infection (types O and A). The Exp group immunized with the test vaccine showed no clinical signs of FMD, and viremia was not detected in oral swabs and sera. However, the other control groups showed clinical signs of FMD, and viremia was detected in oral swabs and sera. Therefore, the test vaccine elicited robust host defense against FMDV infection by modulating a potent host immune system (**Figure 6**).

In a previous study, D-galacto-D-mannan is a dectin-2 agonist that was selected for use in oral vaccines as an adjuvant for FMD vaccines (70). D-galacto-D-mannan can be used in large quantities because it is a natural product with low cost and few side effects. We first evaluated the efficacy of D-galacto-D-mannan as an adjuvant for intramuscular vaccine before evaluating its efficacy as an adjuvant for oral vaccine. However, furfurman is also a dectin-2 agonist that elicits potent immune responses as an FMD intramuscular vaccine adjuvant. In this study, we demonstrated that D-galacto-D-mannan is not the only adjuvant for FMD vaccines but that furfurman elicits potent early, mid-term, and long-term immune responses as host defense as an FMD vaccine adjuvant. However, in this study, only a few cytokines were measured within a narrow vaccination period (7 dpv), and the signaling pathways induced by furfurman were not revealed. In future studies, we intend to overcome these limitations by elucidating the background of the potent immune response induced by furfurman for different vaccination periods. Our study confirmed the efficacy of furfurman as an FMD vaccine adjuvant, which is a milestone in the design of next-generation FMD vaccines. Furfurman is believed to be highly useful in controlling hand-foot-and-mouth disease in humans as well because FMD and hand-foot-and-mouth disease, induced by coxsackievirus A 16 and enterovirus type 71, are similar types of diseases. In addition, furfurman is expected to be applied as an adjuvant to control other viral diseases that require the induction of systemic and mucosal immunity.

## Data Availability

The dataset presented in this article is owned by the authors. Requests to access the datasets should be directed to herb12@korea.kr.
