# Supplementary material for: Next-generation adjuvant systems containing furfurman drives potent adaptive immunity and host defense as a foot-and-mouth disease vaccine adjuvant
Source: Front Immunol. 2024 Dec 11;15:1491043. doi: 10.3389/fimmu.2024.1491043 (PMC11687127; doi:10.3389/fimmu.2024.1491043)
Supplement: Supplementary file 1 [file DataSheet1.pdf]

# *Supplementary Material*

# Next-generation Adjuvant Systems containing Furfurman Drives Potent Adaptive Immunity and Host Defense as a Foot-and-Mouth Disease Vaccine Adjuvant

**Hyeong Won Kim, Seokwon Shin, So Hui Park, Jong-Hyeon Park, Su-Mi Kim, Yoon-Hee Lee, Min Ja Lee***

*** Correspondence:** Min Ja Lee: herb12@korea.kr

## 1 Supplementary Tables and Figures

### 1.1 Supplementary Table

**Supplementary Table 1. List of primer sequences for qRT-PCR.**

| Target | Forward/Reverse | Sequence (5'- 3') | Length (mer) |
|---|---|---|---|
| *ifnα* | *ifnα* F | CATCTGCTCTCTGGGCTGTG | 20 |
| | *ifnα* R | TGAGGGGATCCAAAGTCCCT | 20 |
| *ifnβ* | *ifnβ* F | TGCAACCACCACAATTCCAGA | 21 |
| | *ifnβ* R | GGTTTCATTCCAGCCAGTGC | 20 |
| *ifnγ* | *ifnγ* F | GCCATTCAAAGGAGCATGGAT | 21 |
| | *ifnγ* R | CTGATGGCTTTGCGCTGGAT | 20 |
| *il-1β* | *il-1β* F | AGCCAGTCTTCATTGTTCAGGT | 22 |
| | *il-1β* R | TCATCTCTTTGGGGCCATCAG | 21 |
| *il-6* | *il-6* F | CTGCAGTCACAGAACGAGTG | 20 |
| | *il-6* R | CGGCATCAATCTCAGGTGCC | 20 |
| *il-12p40* | *il-12p40* F | GGAGTATAAGAAGTACAGAGTGG | 23 |
| | *il-12p40* R | GATGTCCCTGATGAAGAAGC | 20 |
| *hprt* | *hprt* F | CCCAGCGTCGTGATTAGTGA | 20 |
| | *hprt* R | GCCGTTCAGTCCTGTCCATA | 20 |

## 1.2 Supplementary Figure

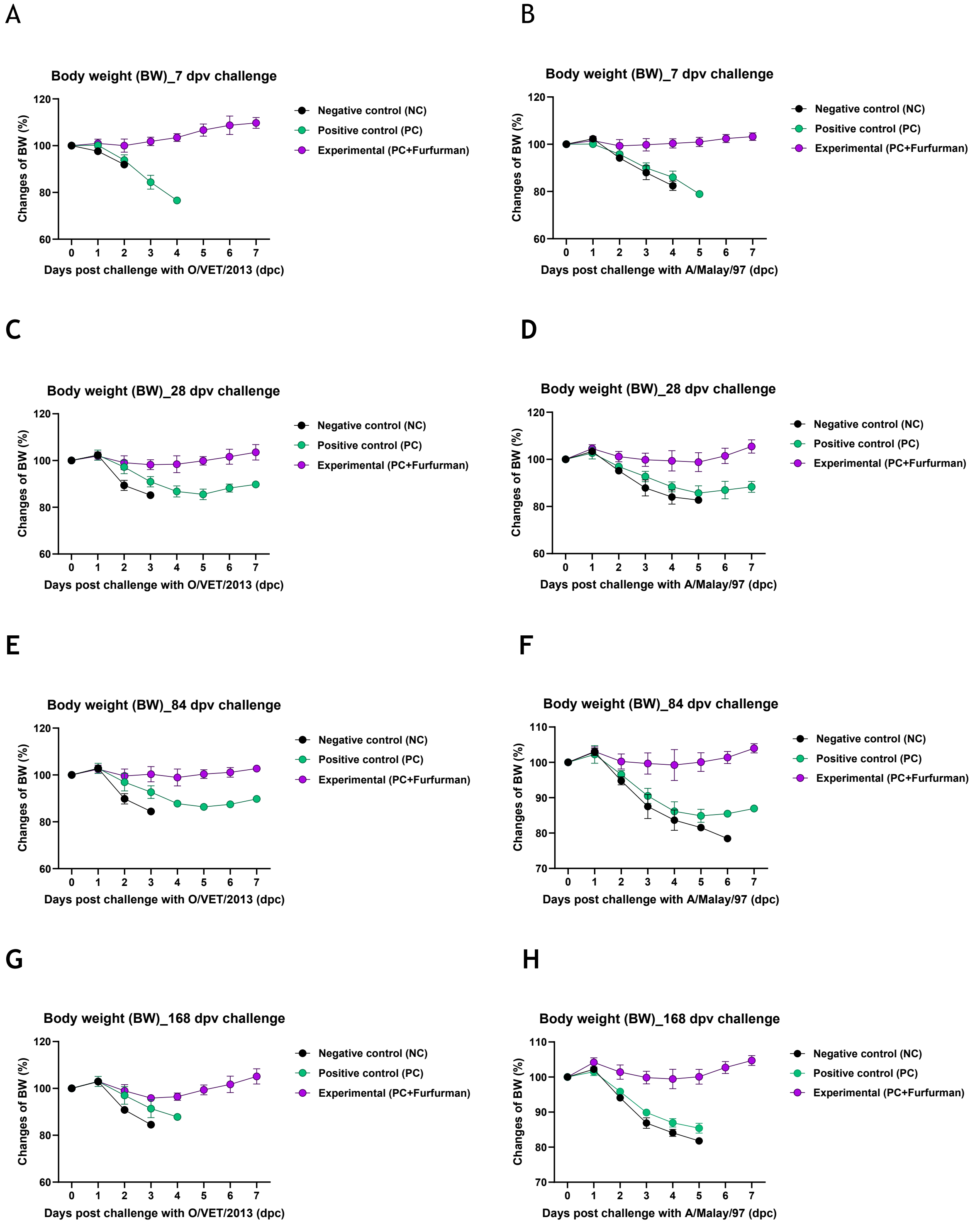

**Supplementary Figure 1. Vaccine containing furfurman induces broad-duration host protection against viral infection in mice.**

C57BL/6 mice were administered the test vaccine with furfurman (Exp group) or without (PC group). The negative control (NC) group was injected with an equal volume of phosphate-buffered saline (PBS). The test vaccines were injected via the intramuscular route into mice that were later challenged with foot-and-mouth disease virus (FMDV) O (100 lethal dose 50% [$LD_{50}$] O/VET/2013) or FMDV A (100 $LD_{50}$ A/Malay/97) at 7, 28, 84, and 168 days post-vaccination (dpv) via the intraperitoneal (IP) route. Survival rates and body weights were monitored for 7 days post-challenge (dpc). (A–H) Body weights post-challenge with O/VET/2013 (A) and A/Malay/97 (B) at 7 dpv; body weights post-challenge with O/VET/2013 (C) and A/Malay/97 (D) at 28 dpv; body weights post-challenge with O/VET/2013 (E) and A/Malay/97 (F) at 84 dpv; body weights post-challenge with O/VET/2013 (G) and A/Malay/97 (H) at 168 dpv. Data are represented as the mean ± SEM of triplicate measurements ($n$ = 5/group).
